# Survey on Ranging Sensors and Cooperative Techniques for Relative Positioning of Vehicles

**DOI:** 10.3390/s17020271

**Published:** 2017-01-31

**Authors:** Fabian de Ponte Müller

**Affiliations:** German Aerospace Center (DLR), Institute of Communications and Navigation, 82234 Wessling, Germany; fabian.pontemueller@dlr.de

**Keywords:** vehicle sensors, laser scanner, GNSS, cooperative, vehicle-to-vehicle, relative positioning, localization

## Abstract

Future driver assistance systems will rely on accurate, reliable and continuous knowledge on the position of other road participants, including pedestrians, bicycles and other vehicles. The usual approach to tackle this requirement is to use on-board ranging sensors inside the vehicle. Radar, laser scanners or vision-based systems are able to detect objects in their line-of-sight. In contrast to these non-cooperative ranging sensors, cooperative approaches follow a strategy in which other road participants actively support the estimation of the relative position. The limitations of on-board ranging sensors regarding their detection range and angle of view and the facility of blockage can be approached by using a cooperative approach based on vehicle-to-vehicle communication. The fusion of both, cooperative and non-cooperative strategies, seems to offer the largest benefits regarding accuracy, availability and robustness. This survey offers the reader a comprehensive review on different techniques for vehicle relative positioning. The reader will learn the important performance indicators when it comes to relative positioning of vehicles, the different technologies that are both commercially available and currently under research, their expected performance and their intrinsic limitations. Moreover, the latest research in the area of vision-based systems for vehicle detection, as well as the latest work on GNSS-based vehicle localization and vehicular communication for relative positioning of vehicles, are reviewed. The survey also includes the research work on the fusion of cooperative and non-cooperative approaches to increase the reliability and the availability.

## 1. Introduction

Advanced driver assistance systems play an important role in increasing the safety and efficiency of today’s roads, while the knowledge about the position of other vehicles is a fundamental prerequisite for numerous safety-critical applications in the Intelligent Transportation System (ITS) domain. Safety-critical applications, as for instance Forward Collision Avoidance (FCA), Lane Change Assistance (LCA) or Automatic Cruise Control (ACC), need continuous knowledge about the relative position and relative velocity of other vehicles in the vicinity of the ego vehicle.

For almost a decade, relative positioning sensors, such as radar sensors, have been available in commercial vehicles. In the last few years, camera systems have found their way into high-end vehicles for collision avoidance, lane-keeping assistance and in-vehicle traffic sign recognition. The first prototypes of fully-autonomous vehicles use 3D laser scanners to obtain an accurate representation of the surrounding environment. The richness and high precision of these devices makes it possible for autonomous vehicles to obtain a detailed representation of the scenery including the exact position of buildings, vegetation, other road participants and further obstacles. In this way, the robotic vehicle is able to self-localize itself and navigate through traffic [1].

With the standardization of the first Vehicle-to-Vehicle (V2V) communication protocols in Europe, America and Japan, cooperative approaches have made it possible to extend the perception range of the ego vehicle beyond the capabilities of on-board ranging sensors by using information from other vehicles in the surroundings. The European Telecommunications Standards Institute (ETSI) and the U.S. Society of Automotive Engineers (SAE) are currently working on the definition of different safety-critical messages for the V2V technology. Each vehicle will transmit periodically Cooperative Awareness Messages (CAMs) [2] or Basic Safety Messages (BSMs) [3] containing basic information, such as position, speed and heading. The included position in global coordinates can be used by a vehicle to estimate its neighbors’ positions. Its own coordinates might be estimated using a Global Navigation Satellite System (GNSS), like the American Global Positioning System (GPS) or the European Galileo system. This estimate can be additionally enhanced by supporting it with on-board sensors, such as wheel angle, odometer and inertial sensors.

Although it is demonstrated that autonomous road vehicles can rely solely on their on-board perception sensors, it is foreseen that they will greatly profit from the introduction of an inter-vehicle communication. Besides an increased availability and reliability in cooperative relative positioning, the communication enables cooperative perception by sharing sensor information and the execution of collaborative maneuvers between automated road vehicles. In this way, a higher degree of safety is achievable without sacrificing efficiency by driving with large safety distances and increased caution.

**Figure 1:**
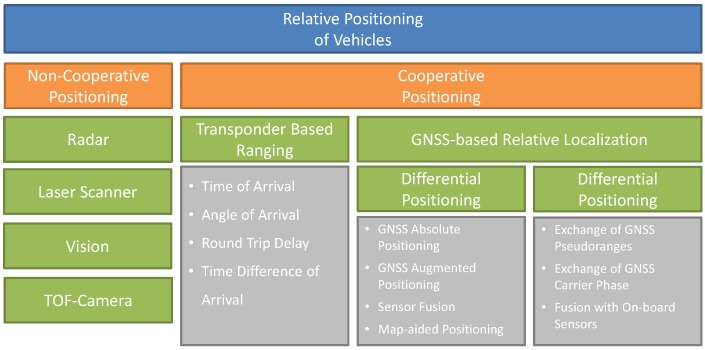
Technologies for relative positioning of vehicles. In this work, we differentiate between non-cooperative and cooperative solutions.

A first overview of the different technologies and methods for relative vehicle localization is shown in Figure 1. The aim of this work is to present commercially-available solutions for the relative positioning of vehicles and to give a sound review on the latest research contributions in this field. Hence, this paper will focus on both non-cooperative and cooperative solutions. The performance, advantages and disadvantages of each technology will be discussed in detail.

As a first step, in Section 2, the requirements from the applications on vehicle relative positioning systems will be named and quantified. Section 3 gives a comprehensive review on non-cooperative ranging sensors that are already commercially available or currently under research. Then, Section 4 focuses on cooperative approaches for relative vehicle positioning based on the exchange of information between road participants. In Section 5, different approaches of fusing the information of different non-cooperative sensors or coupling the information of both cooperative and non-cooperative solutions are discussed. The Conclusions discusses the different approaches that were presented, synthesizing their advantages and disadvantages.

## 2. Requirement Analysis

In this work, the ego vehicle is defined as the vehicle that requires information on the position of surrounding vehicles with respect to it. The surrounding vehicles, the target vehicles, are located around the ego vehicle, possibly in another lane, another direction or street. It can also happen that the relative positioning system might detect vehicles driving on another level, as for instance on an overhead bridge.

**Figure 2:**
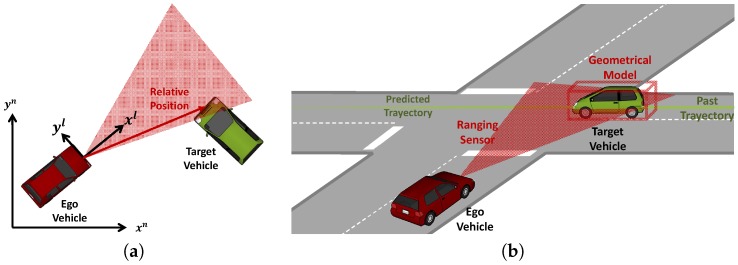
The figure (**a**) depicts the ego and target vehicle driving in an Earth-fixed navigation frame $\left[x^{n},y^{n}\right]$. A ranging sensor in the ego vehicle is able to detect the target vehicle, whose position is given in an ego vehicle coordinate frame $\left[x^{l},y^{l}\right]$. The figure (**b**) shows how a geometric model of the target vehicle can be constructed from the ranging sensor measurement data. Using a tracking algorithm, the future location of the target vehicle can be predicted.

The relative position of a target vehicle as seen from the ego vehicle is generally supplied in the ego vehicle coordinate frame. In automotive applications, usually a coordinate system is defined at the foremost center point of the vehicle with the *x*-axis pointing in the driving direction, the *y*-axis perpendicular to it in the left direction and the *z*-axis pointing up. The relative position is, in general, the three-dimensional vector pointing from the origin of the ego coordinate system to a predefined point in the target vehicle. Since vehicles generally drive on the same plane, a two-dimensional relative position is provided by many systems and is sufficient for most safety critical applications. This is depicted in Figure 2a. Many driver assistant applications require, along with the relative position, information about the relative velocity towards surrounding vehicles. For instance, FCA applications profit from having a measurement of the relative speed in order to predict more accurately the Time to Collision (TTC) with another car.

In order to compare non-cooperative and cooperative approaches, the way in which relative position information in each of the technologies is obtained has to be explained. Radar, laser scanners and vision-based systems first perform the measurement step, in which raw measurement data are extracted out of a raw signal by applying signal processing algorithms. The raw measurement data are a cloud of reflecting points in radar and laser scanners or one or several pixel matrices in vision-based systems. In the detection step, the raw measurement data are segmented and clustered into objects. An object classification step aims at sorting objects into different classes, as for instance vehicles, bicycles and pedestrians. Usually a geometric model is used to describe objects in a parametrized way. Vehicles are thus abstracted as rectangular cuboids, which are described by its position, its dimension and its orientation in the ego vehicle coordinate frame (see Figure 2b).

After the detection, the position of the vehicle needs to be estimated over time. This is known as tracking and can be performed in many different ways. Usually, movement models that predict the future path of the vehicle are used. Common movement models range from simple kinematic models as the rectilinear-constant-velocity model or the constant-accelerated-constant-turn-rate models, to dynamic models that take forces into account, as the well-known two-wheel bicycle model or the four-wheel Ackermann model. New measurements are included by performing a data association step in which the target that most probably produced the measurement is found. In the last step, a semantic classification provides a broader understanding of the surrounding situation and enables higher level reasoning, as for instance to control an automated vehicle. Further information regarding the different processing steps in object detection and tracking for vehicular environments can be found in [4,5,6].

Cooperative approaches based on V2V communication differ from this processing chain in the way that position information is made directly available at the ego vehicle. The measurement, detection and classification steps disappear. Object tracking and filtering are still necessary due to the varying uncertainty in the communicated information and the incompleteness of the data due to communication outages. Next, the requirements on relative positioning are listed.

### 2.1. Accuracy

In general, the accuracy of a measurement system could be defined as “how close” the measurement is to its true value. This closeness is quantified by the distance between the measured and true value. In positioning systems, accuracy is usually given in three dimensions. Especially in applications where the vertical position is less relevant, as in vehicular applications where cars drive on roads, the 2D accuracy is used instead. Position accuracy is usually quantified by the Root Mean Squared Error (RMSE) or other conventions as the 95% confidence interval. When evaluating the accuracy of relative positioning systems for vehicles, it is common to differentiate between along-track and across-track accuracy. Especially in on-board ranging sensors, a differentiation between range accuracy and angular accuracy is made. Along with the relative position and relative velocity, it is useful for driver assistance applications to have information about the current uncertainty in each of the measurements. Sensor systems might have different precisions depending on the range towards the target vehicle, the view angle, the material the target is made of or the environmental conditions. A relative positioning system would ideally provide a highly accurate and bias-free relative position and relative velocity output in both the along- and across-track direction along with a measure of the current uncertainty of its estimation. Shladover and Tan stated that 1 m position accuracy could be marginally acceptable for collision warning applications, while 50 cm would yield significantly better results [7].

### 2.2. Reliability

Especially for safety-critical applications, the system’s reliability is a very important factor. The accuracy of a system might be very high, but if it is not reliable, it cannot be counted on. In system engineering, reliability is also called integrity. Integrity gives “a measure of the trust which can be placed in the correctness of the information supplied by the total system” [8]. Integrity also includes the ability to provide alarms when the error tolerance of a certain parameter is exceeded. Integrity analysis yields a confidence interval for a certain parameter, a so-called protection level, along with the integrity risk, i.e., the probability that a measurement is not contained within the protection level.

While in civil aviation stringent integrity requirements are imposed on all involved operational components, in the field of road transport, integrity is starting to be specifically considered. The Functional Safety for Road Vehicles standard (ISO 26262) defines the so-called Automotive Safety Integrity Levels to quantify the risk associated with each of the function, software and hardware components inside a vehicle and related to safety critical applications [9].

### 2.3. Availability

It is of high importance that a positioning system is as available as often possible. Multiple causes can be the reason for a diminished availability. A GNSS-based positioning system might not be available in situations with complete obstruction in the line-of-sight to the satellites, e.g., in tunnels. Not only GNSS, but any type of radio-based positioning system can be made unavailable by jamming the signal. Vision-based ranging systems might not be available in adverse whether conditions, such as fog, heavy rain or at night. Cooperative approaches are only available if each of the vehicles is equipped with the required localization and communication devices. At the early development stages, the low penetration rate will yield a de facto low availability. Hence, in order to provide a continuous operation of a safety system, the requirement on availability should reach a value near 100%.

### 2.4. Detecting Range and Field of View

Some ranging systems, as for instance laser scanners or visual systems, have line-of-sight characteristics, meaning that they can only measure the position of neighbors, to whom they have direct visibility. They can be easily obstructed by other vehicles, buildings or the surrounding topography. These systems have also a technical range limitation, related to the limited transmit power or sensor sensitivity. Additionally, they also have a limited field of view, defined by the opening angles in azimuth and elevation in which they can scan the environment. To overcome this limitation, multiple instances of a sensor need to be placed around the vehicle to get a 360 degree view of the environment. With V2V communication and omnidirectional antennas, an all-around perception of the environment is achieved.

### 2.5. Dimension

A position in space is a three-dimensional component, so it is a relative position coordinate. However, many relative positioning systems are only able to measure the relative position in one or two dimensions. A laser scanner uses a rotating mirror around the vertical axis to scan the environment in the azimuth angle. Consequently, it can output a two-dimensional relative position while disregarding the vertical component. An Ultra-Wideband (UWB) system, on the other hand, is able to measure the Round-Trip Delay (RTD) of signals emitted from the ego vehicle and returned by other vehicles. This system is only able to estimate the range between the vehicles, but not the exact two- or three-dimensional position. Cooperative solutions based on GNSS are able to deliver a full 3D relative position between vehicles.

### 2.6. Target Resolution and Identification

The resolution of targets is the ability to resolve different objects. This is important for driver assistance systems to quantify the number of targets and to track them over time. Nearby located targets can be erroneously merged by on-board ranging sensors into one single object. The processing capabilities inside ranging sensors and on-board the vehicle are constrained, and consequently, the maximum number of detected and tracked targets is limited. Depending on the road environment, it will be required to track up to 100 targets in order to obtain the required awareness for safety-relevant applications. Additionally, the unambiguous identification of targets over time is a further requirement for relative positioning solutions. This involves giving the same target the same ID when its detection and tracking were interrupted.

### 2.7. System Delay

For safety-relevant driver assistance applications, a fast response and high dynamics are required. This is important for a warning system to give timely alarms and for a controlling system be able to respond smoothly to changes in the relative position. For this, the information from the relative position device has to be processed in a short time period and be output at a sufficiently high rate. In platooning, a fast and reliable control of the speed and steering of the vehicle is pursued [10]. While today’s ACC applications require between a 10 Hz to 20 Hz update rate [11], future collision detection and pre-crash applications require update rates up to 50 Hz [12].

The output rate of measurements is one important factor. However, the delay in the measurement, i.e., the time elapsed since a physical event occurs until it is output to the application, is significant, since it causes a delayed detection for FCA or an unstable controlling in platooning applications. For instance, radar sensors and laser scanners are not sensitive to changes in velocity. Thus, they can only estimate acceleration by looking at consecutive measurements and, consequently, suffer from an increased delay. A cooperative solution that directly transmits sensor information from the vehicles will overcome this limitation. However, a propagation and communication system delay will be introduced by the V2V communication [13].

### 2.8. Non-Technical Requirements

There are other non-technical requirements or limitations that should be taken into account when evaluating the suitability of a certain relative positioning system. In commercial passenger and freight vehicles, the price plays an important role. The cost of a certain relative positioning solution is not only the direct price of the device that needs to be equipped in the car, but also secondary costs need to be considered, including installation and maintenance costs, processing power, weight and power consumption, noise and heat creation. In cooperative solutions that use infrastructure-based communications, such as cellular communication, running costs in the form of monthly or annual fees for maintaining the infrastructure and the use of the licensed frequency band can be expected.

## 3. Non-Cooperative Relative Positioning

This section focuses on egocentric or non-cooperative approaches, while the following section analyzes cooperative approaches. Egocentric techniques are passive or non-cooperative in the way that a target vehicle is not actively contributing to the estimation of its relative position.

### 3.1. Radio Ranging

One simple technique to estimate the range towards another vehicle consists of using electromagnetic waves and measuring the Received Signal Strength (RSS) of a signal transmitted by another vehicle. This is considered as a non-cooperative approach, since the signals are not transmitted for the primary purpose of localization. Such signals are generally known as signals of opportunity [14]. The signal level is proportional, under ideal conditions, to the distance between the transmitter and receiver antenna. By assuming a certain model for the power decay over distance, given by the path loss exponent, a rough estimate of the range between two cars can be performed. Since V2V communication technology is foreseen to be integrated in future vehicles, several research groups have analyzed the possibility of performing RSS ranging with this technology. Alam et al. use Doppler measurements in [15] to estimate the path loss exponent. Their simulated distance estimation achieves an accuracy of 5.7 m at high relative speeds. In [16], Parker et al. propose a cooperative technique that uses RSS in combination with speed measurements from the vehicles in a cluster. Although they demonstrate theoretically that their approach is accurate, reliable and can be real-time, they do not reveal the measurement uncertainty used for the RSS measurements. This is a key issue in RSS, since real-world experiments show that only a rough estimate of the user position can be acquired in this way, since shadowing and multipath cause large variations in the received signal strength. Additionally, the RSS method is very sensitive to how well the path loss exponent is estimated [17]. In [18], errors above 20 m are reported for short distances between vehicles. A technology based on RSS-ranging is only able to offer a distance towards the target vehicle. To overcome this shortage, Lie et al. propose a multiple antenna relative localization system based on RSS that achieves 98% accuracy in locating another vehicle in the correct lane.

Radar is a technology that uses high-frequency electromagnetic waves to measure the distance and relative speed of target objects. Radar sensors can already be found today in middle class vehicles for forward collision warning applications, lane change assistance or automatic cruise control. Two main radar technologies exist in ITS. Impulse radar, on the one hand, measures the time needed for a short pulse to travel from the radar sensor to the object, reflect and travel back to the sensor. On the other hand, Frequency-Modulated Continuous Wave (FMCW) radars transmit a frequency-modulated signal with a constant power envelope. The frequency difference between the outgoing and incoming waves is directly proportional to the relative distance to the target object. Both radar types are capable of measuring the relative speed by exploiting the Doppler effect and looking at the frequency difference between the outgoing and incoming waves. Table 1 lists the characteristics and performance parameters for five commercially available radar sensors taken from the respective manufacturer’s data sheet.

Radar sensors work typically in two frequencies. On the one hand, the 77 GHz band is used for so-called long-range radars [19]. On the other hand, the 24 GHz UWB band is temporarily allowed in Europe for automotive short-range radar sensors. Also in this band, narrowband radars can be operated using the 100 MHz Industrial, Scientific and Medical (ISM) band. At 24 GHz, radars coexist with radio communication devices and radio astronomy stations that might be impaired if too many automotive radars are operated. Therefore, the European Commission has made available the 79 GHz band for long-term operation of radar sensors seeking worldwide harmonization. This band has the advantage that a common technology for short-range and long-range radars can be developed, along with the decreased dimension and weight and increased Doppler sensitivity [20]. The usage of the 24 GHz UWB band has been prolonged until January 2018 [21]. However, mutual interference of radar sensors is also becoming a growing issue in radar technology [22,23].

Typically, a distinction is done between short-range radar up to 20 or 30 m, medium-range radar up to 100 m and long-range sensors that measure beyond this distance up to 250 m [24]. Short-range radars are used for collision avoidance and object detection and need higher range resolution and, consequently, more bandwidth (4 GHz at 77 GHz to 81 GHz). They usually have also a wider field of view up to ±30${}^{\circ }$. Long-range radars, on the other hand, are mainly used for ACC, can live with a lower range resolution and are designed with smaller bandwidths (500 MHz between 76 GHz and 77 GHz). Their field of view is usually smaller and around ±10${}^{\circ }$. In general, the range and speed resolution of a radar is determined by its bandwidth. Products available on the market state range accuracies of 10 cm up to 1% to 5%, while the speed accuracies are around 0.2 m/s.

Generally, the across-track accuracy of radars is low. The approach used to estimate the angle of arrival of the reflected signal is to implement an array of patch antennas and compare the amplitude and phase of signals in partially overlapping beams. Some radar sensors provide moderate angular resolution by using mechanical sweeping radar scanners [19]. Angular accuracies between 0.5${}^{\circ }$ and 5${}^{\circ }$ are typical, yielding lateral uncertainties from 87 cm–4 m at 50 m, respectively.

Targets can be easily resolved when they are located in different ranges and velocity cells [25]. For instance, commercial radar sensors claim to have a correct resolution of objects when their distance difference is above 1 m or their speed difference is above 2 km/h. The target resolution for objects at approximately the same distance and speed relies on lateral resolution, which is dependent on the lobe width, which, at the same time, depends on the wavelength. Larger wavelengths yield broader lobes or the same antenna dimension [26]. Therefore, moving from 24 GHz to 79 GHz yields a better lateral resolution and the availability of the 4 GHz bandwidth enhances the overall longitudinal resolution of target range and velocity.

Other characteristics of radar sensors are their invisible integration behind electromagnetically-transparent materials, as for instance, behind the front bumper. Besides a few exceptions, radars usually have no moving parts and are, therefore, more robust and less prone to mechanical failures than laser scanners. In contrast to vision-based and laser scanners, radars are robust against environmental conditions, such as changes in light or fog and rain. In recent years the price of radar sensors has dropped substantially, and they are available in middle and high class vehicles.

### 3.2. Laser Scanners

Light Detection and Ranging (LIDAR) devices are laser-based ranging systems that, similar to radar, are based on the time-of-flight of reflected light pulses and are able to measure the distance towards an object. They usually work in the near infrared region of the electromagnetic spectrum at 905 nm. Their transmit power is limited to complying with eye-safety regulations, which imposes a practical limit on the measuring range of the sensor. Usually, a rotating device makes it possible to use a few laser light sources to scan points in space. These devices are called laser scanners. 2D laser scanners measure points in one plane, while 3D laser scanners are able to also vary the elevation angle. Many available products use different scanning planes in order to achieve a small range of elevation angles to account for the inclination of the vehicle.

The use of laser scanners is mainly in the field of obstacle detection, collision mitigation and stop-and-go assistance. Nevertheless, the use of laser scanners for ACC is already finding its place in ITS. Future autonomous cars will likely rely on laser scanner information to get information from surrounding obstacles [27]. Unfortunately, laser scanners are not able to measure the relative speed between ego and target vehicle directly, but differentiating the range of successive scans [27].

Table 2 summarizes the performance of four commercially available laser scanners in terms of dimensional resolution, maximum range, azimuth angle, accuracy and measurement cycle. The accuracy is divided into range, radial speed and azimuth angle. As the output power of automotive laser scanners is limited, their maximum perception range lies between 80 m and 200 m and strongly depends on the light reflectivity index of the object. Short-range laser scanners for object detection have ranges of around 10 m. With values around 100${}^{\circ }$, the field of view of laser scanners is generally broader than that of long-range radar sensors. Even 360${}^{\circ }$ laser scanners that scan the whole surrounding at high revolutions with several laser beams exist. These have been used in autonomous vehicles as Junior from Stanford University or the Google self-driving car.

Laser scanners feature good range accuracy between 0.02 m and 0.5 m. The lateral accuracy is limited by their angular resolution of around 0.1${}^{\circ }$. The speed accuracy of 0.5 m/s is slightly worse, but comparable to that of radar sensors. The availability of laser scanners is importantly limited by environmental conditions. Fog, rain, dust, dirt and water heavily detriment the performance of the sensor. Furthermore, incident sunlight in the morning and afternoon hours can cause important disturbances on the laser detecting device. The price is still high for laser scanners being incorporated into commercial vehicles. Nevertheless, they are extensively used by many research groups to test novel advanced driver assistant systems or self-driving vehicles.

### 3.3. Time-Of-Flight Cameras

Time-Of-Flight (TOF) or 3D cameras are used in many different fields, including human-machine interaction, industrial automation and robotics. In automotive applications, they are used in driver assistance and safety applications, such as pedestrian recognition or pre-crash detection [28]. Unlike laser scanners, the TOF camera captures the entire scene with one single light pulse. Each camera pixel measures the time delay of modulated infrared light by comparing the phase of the outgoing and the incoming signal [29,30]. The distance information is captured simultaneously for the entire scene [31].

When compared to video cameras, the TOF camera’s CMOS sensors have currently much lower resolution (64 × 8 pixels [31] and around 200 × 200 pixels in Table 3). The typical range up to where TOF cameras operate is 10 m and, therefore, suited for pedestrian recognition, parking assistance or object recognition. Elkhalili presents a CMOS sensor for an TOF camera that targets a distance of about 20 m [31]. The field of view of TOF cameras is around 40${}^{\circ }$.

A typical value for the ranging accuracy is around 1 cm. Compared to video-based techniques, TOF cameras use a single lens and reach more accurate depth information [28]. When compared to laser scanners, TOF cameras have higher frame update rates ranging from 20 fps [32] to 200 fps [31]. The advantage of TOF cameras is that they do not use mechanical components as laser scanners. TOF camera sensors, like laser scanner sensors, have problems with incident sunlight, which is a great drawback for situation awareness in automotive applications. Additionally, the price of TOF cameras, although decreasing in recent years, is still high for market introduction.

### 3.4. Vision-Based Solutions

Machine vision techniques are able to detect and localize objects by processing the images drawn from an imaging device like a camera. Although vision can provide highly valuable information about the environment, image processing techniques are complicated, computationally expensive and still under research. For automotive vision sensors, processing of road scenes can provide accurate information other sensors fail to obtain. Already today, cameras are being introduced in high-class vehicles for detecting lane marks and offer lane keeping assistance or lane departure warning systems. Furthermore, automatic traffic sign recognition systems are already able to inform the driver about the current speed limit and other types of hazards along the road. Further on, cameras are recently incorporated for object detection. Especially the detection of pedestrians, which would otherwise fail with radar sensors or laser scanners, can be accomplished with vision-based solutions.

Generally, for a camera system, it is relatively easy to measure distances in the lateral, cross-track, direction. Depending on the resolution of the camera sensor, a certain distance-dependent lateral accuracy can be obtained. Vision processing is less effective for calculating the longitudinal, along-track, distance of an object. Monocular systems use one sole camera and exploit the geometry of the scene, along with the knowledge about the size of the objects, in order to estimate the longitudinal distance. On the contrary, in stereo-vision-based approaches, two cameras are able to directly estimate the 3D coordinates of an object. A survey on object detection algorithms for stereo cameras can be found in [33], while [34] gives a sound review on pattern analysis for vehicle detection. A comprehensive overview and literature survey on monocular and stereo-vision vehicle detection techniques including tracking and behavioral modeling is presented by Sivaraman et al. [35]. In their work, vehicle detection using vision is briefly compared with complementary technologies, as for instance radar and LIDAR. The use of the occupants’ smartphone camera for vehicle distance estimation has been proposed in [36,37,38]. However, the inexpensive camera technology and the limited processing capabilities might, depending on the smartphone model, impose limitations on the performance regarding relative position accuracy, reliable detection of vehicles and the number of tracked objects.

Camera systems have sensing ranges from 4 m–80 m and a field of view of ±20${}^{\circ }$ in azimuth [39]. However, stereo-vision and depth capability rapidly drop with distance and are limited to a range of up to 40 m [40]. In [41], the authors propose a 360${}^{\circ }$ vision-based system with three cameras around the vehicle.

While radar and laser scanners have rather constant ranging errors over distance, the range accuracy of camera systems typically decreases quadratically with distance [42]. At 3 m, a range error of 5 cm is stated in [43], while the error at 40 m is around 3 m. Nedevschi et al. achieve an accuracy of around 1% at ranges from 10 m to 95 m with a stereo camera system [44]. Huval et al. apply deep learning techniques with a monocular camera to achieve a 6 m range error up to a 60 m distance [45]. The error increases to 20 m at 120 m.

Stein et al. have concluded in [46] that a monocular vision sensor offers sufficient accuracy in range and range rate to be used in an ACC application. By using the laws of perspective, they state range errors of 5% at 44 m and 10% at 90 m. Furthermore, Giesbrecht et al. worked on a monocular vision system for a robotic follower vehicle obtaining a mean distance error of 0.62 m for distances of 10 m to 23 m [47]. With an approach based on fusing a priori knowledge of the vehicle width using a vehicle type classifier with geometric constraints of the street, Leßmann et al. achieved an accuracy of 6.5% for ranges of 5 m to 80 m with a monocular vision system [48].

Cameras work usually at frame rates between 15 fps and 25 fps and, thus, have measuring rates comparable to radar sensors and laser scanners. However, relative velocity cannot be directly measured, but has to be differenced from successive images. Camera sensors are, like human visual perception, sensitive to adverse lighting conditions, as for instance, fog and rain or low sun and blinded by the head lights of approaching vehicles.

## 4. Cooperative Relative Positioning

**Figure 3:**
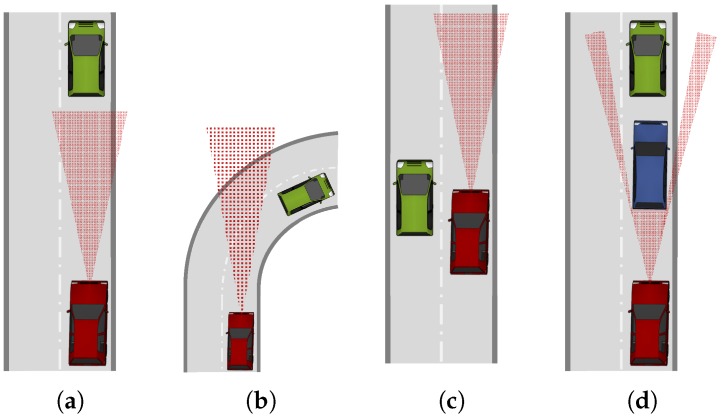
The four figures depict situations where a ranging sensor at the red ego vehicle has a limited performance in estimating the relative position of the green target vehicle. Either because of a limited sensing area in terms of range (**a**), or aperture (**b**,**c**), or due to an obstacle that blocks the line-of-sight (**d**).

A series of non-cooperative approaches has been presented in the previous section, with radar, laser scanners and vision-based systems being the most important technologies. A common problem of these three sensor systems is their line-of-sight characteristic. As shown in Figure 3, there are many situations in which these sensors are not able to offer a relative position and velocity estimate. The limited range, the limited field of view and sight blockage by other vehicles or the surrounding topography are common situations. The underlying problem is the high frequencies in the radio spectrum where these three technologies work and their inherent propagation characteristics. Millimeter waves used in radar frequency bands behave nearly similar to the optical frequencies of laser scanners and vision-based solutions. They are mainly line-of-sight and are blocked by cars or buildings. For this reason, the following approaches are able to extend the awareness range of the ego vehicle by using lower frequencies.

This section presents cooperative positioning techniques where two or more mobile stations work together to improve their position solutions communicating directly with each other. Now, target vehicles are no longer passive, but have some sort of device installed that is for the sole purpose of making other vehicles aware of it. As shown in Figure 1, cooperative positioning techniques can be divided in transponder-based ranging systems, which estimate the relative position directly at the RF-signal level, and GNSS-based relative localization techniques, where GNSS-related information is directly exchanged between the vehicles using a dedicated communication technology.

### 4.1. Transponder-Based Ranging

One possibility is reusing the idea of radar at lower frequencies. However, due to the larger wavelength, vehicles appear rather small and do not offer a sufficiently big surface to reflect radio waves. Therefore, an antenna is installed on a target vehicle and the signals are amplified and “reflected” back to the transmitter in the ego vehicle. The relative distance of the target vehicle can be estimated by measuring the RTD and multiplying it by the speed of light.

Radio ranging based on UWB technology has been part of the work of the research group around Petovello [49]. Here, a UWB signal at 6.35 GHz is used for estimating the range towards other vehicles with decimeter-level precision up to 300 m of distance. The two-way ranging technique used in their work is assessed in [50]. Unlike Petovello, Morgan used 2.4 GHz and 5.9 GHz radios to estimate the range towards a set of road side units in order to perform Time-Difference-Of-Arrival (TDOA) and absolute position [51]. He concluded that ranging performance using V2V technology is highly dependent on relative velocity and distance. In his test setup, he measured ranging errors of up to 0.7 m at distances up to 90 m in line-of-sight situations. Furthermore, Staudinger and Dammann performed RTD measurements at 5.5 GHz with LoS and multipath-rich environments obtaining accuracies between 0.5 and 1 m [52]. Perker et al. performed simulations for absolute positioning fusing GNSS and RTD measurements from several vehicles in [53]. The main problem with RTD is that the delay caused by the target vehicle to reflect the signal back has to be estimated. In [53], the solution was to use physical level timestamping at reception and retransmission. Other limitations to the accuracy are imposed through multipath propagation and, especially, non-line-of-sight propagation.

Usually, radio ranging systems based on RTD will output an estimate of the range towards the target, but not a complete 3D relative position by an estimate on the bearing angle or the elevation angles. For this, Angle-Of-Arrival (AoA) techniques are required, where, using multiple antenna elements, the direction of arrival of the wave can be estimated [54].

Other transponder-based solutions rely on measuring the time the signal takes to travel from the transmitter to the receiver; techniques usually known as Time-Of-Arrival (TOA). This, however, has the drawback of requiring precisely synchronized clocks at every vehicle, in order to compare the transmission and reception timestamps [17]. In order to obtain decimeter-level ranging accuracy, a transceiver clock synchronization accuracy of less than 0.3 ns is required in each transceiver. Blumenstaien and Vychodyl investigated TOA measurements in UWB and millimeter wave for inter-vehicle ranging achieving decimeter-level ranging accuracy [55,56]. The problem of clock unsynchronization, however, falls out of the scope of their work.

### 4.2. GNSS-Based Relative Localization

With the introduction of a comprehensive and standardized communication technology for ITS, the idea of performing cooperative positioning in vehicular ad hoc networks becomes a reality. Unlike the previous techniques, GNSS-based relative localization relies on the information sent over this communication link. In V2V communication, CAMs and BSMs are beacon messages that include the information about the vehicle’s absolute position in Earth coordinates, its speed and heading along with their associated uncertainty. ETSI’s standard for CAM messages does not specify the technique used for computing this information in each vehicle [2]. Most certainly, GNSS will be used as the primary source for position information. Therefore, in this section, we review the different techniques related to GNSS to localize a vehicle. Moreover, we will differentiate between absolute positioning, where a vehicle aims at localizing itself on the Earth, and relative positioning, where the aim is to know the relative position between two vehicles.

#### 4.2.1. Absolute Positioning

By using multiple constellations in parallel, as for instance the American GPS, the Russian GLONASS or the European Galileo system, more satellites can be tracked and used for localization, velocity and time estimation, and thus, an increase in availability and accuracy can be achieved [57]. Further on, the usage of multi-frequency GNSS receivers can help to estimate the delay of the dispersive parts of the atmosphere. Besides the L1 and L2 frequency bands in early GPS, the new L5/E5 band will enhance this capability [58]. However, dual- or multi-frequency receivers are usually more expensive and are usually not used in mass-market applications.

A technique, which has been known since the early years of GPS and is able to reduce atmospheric and other common errors, is differential GNSS (DGPS). In DGPS, a receiver located at a known position (base station) can determine the offset on the range towards each satellite and send this information to other receivers (vehicles) in the vicinity. By correcting this offset prior to position computation, the vehicles are able to obtain a better absolute position. In general, Ground-Based Augmentation Systems (GBAS), as for instance DGPS, use base stations at known locations to transmit real-time correction data for GNSS absolute positioning. The transmission is accomplished using some communication technology, as for instance a dedicated radio communication link or a cellular communication system. When GNSS correction data are transmitted from geostationary satellites, these are referred to as Satellite-Based Augmentation Systems (SBAS). For instance, the American Wide Area Augmentation System (WAAS) or the European European Geostationary Navigation Overlay Service (EGNOS) provide wide area coverage of differential corrections. The general rule applies that the nearer the base station, the more correlated the errors between the base station and receiver are and the larger the improvement of the position solution can be expected. This way, atmospheric errors can be corrected, in general, with base stations up to 20 km [58].

Further on, vehicles also apply sensor fusion techniques to support the GNSS position solution with on-board sensors in GNSS-detrimented environments (under dense tree canopy or urban canyons) or GNSS impaired scenarios (in tunnels). Commonly-used on-board sensors for absolute positioning in vehicular environments are wheel speed sensors, odometers, steering wheel position, accelerometers, barometers and magnetic compasses [59]. The fusion of GNSS with an Inertial Navigation System (INS), composed of two triads of accelerometers and gyroscopes, is a well-known technique for supporting GNSS in aircraft, missiles and ships [60]. In the last decade, these techniques have been extended to vehicle positioning by moving from high-grade and expensive ring laser and fiber optic gyroscopes to low-cost inertial MEMS sensors [61]. The performance of vehicle positioning in challenging environments with various low-cost in-vehicle sensors is addressed in [62,63].

Hence, the topic of absolute positioning for vehicles has been extensively researched in the past two decades. Enhancing GNSS to mitigate the influence of multipath has been explored in [64,65,66,67,68]. Fusion of GNSS with on-board sensors has been addressed by [69,70]. Augmenting GNSS with opportunistic signals, such as cellular networks or WiFi access points, has been analyzed in [71,72,73]. Skog and Händel authored a comprehensive survey on vehicle localization technologies including GNSS and RF-based positioning fused with motion sensors, vehicle models and road maps [59]. Traditional on-board ranging sensors can also be used for absolute vehicle positioning by using a map of the surrounding static objects. The fusion of GNSS with either laser scanners [74,75], radar sensors [76,77] or vision-based systems [78,79,80] offers an increase in robustness and availability when GNSS signals are impaired. An enhancement of GNSS positioning with maps has been developed in [81,82,83]. Last, but not least, cooperative approaches for deriving absolute positions have been proposed in [84,85,86,87,88]. In all of this work, the objective is to estimate the position of the ego vehicle in absolute Earth coordinates.

#### 4.2.2. Relative Positioning

Approaching the problem of relative positioning, in which the position towards other vehicles is the aim, from a cooperative perspective has experienced less attention. Probably due to the ever-growing availability of precise and less expensive ranging sensors, the usage of GNSS for relative positioning has been relegated to second place. The aim in GNSS-based relative positioning is to extend the limited perception range of on-board ranging sensors, while at the same time providing accurate relative position and velocity in an ego vehicle frame meeting the stringent requirements of safety-critical driver assistance applications regarding availability and reliability. Using the information contained inside CAMs or BSMs, it is possible to locate another vehicle in relation to the ego vehicle in the way a ranging sensor would do. This vehicle can thus be modeled with a rectangular cuboid, with the correct vehicle dimensions and oriented on the 2D plane with the relative heading in relation to the ego vehicle.

Röckl et al. have analyzed the use of GNSS absolute positions and speed contained in CAMs to estimate the relative position to other vehicles using a particle filter in [26,89]. In [90], Kellum identified the effectiveness of sharing absolute positions using low-end, mid-range and high-end GNSS receivers and comparing the output to a radar sensor. He concluded that low-cost GPS receivers are not capable of meeting the requirements of safety-critical applications as they showed longitudinal errors of around 2 m and lateral errors above 3 m while driving in a highway environment.

#### 4.2.3. Relative Positioning with Raw GNSS Measurements

The concepts of differential GNSS can be extended by moving from the classic base-station/vehicle setup for absolute position towards a vehicle/vehicle setup for relative position estimation. By sharing pseudorange measurements over V2V communication, each vehicle can subtract the neighbor’s pseudoranges from its own and compute, in this way, a relative position, while at the same time reducing the correlated GNSS errors common to both vehicles. Richter et al. introduce a relative localization approach on the basis of the exchange of GNSS pseudorange data in [91]. They present the concept, but do not present concrete simulations or measurements. In [92], Alam et al. present a tight integration approach, where pseudorange observations from two vehicles are subtracted from each other, and show its theoretical potential through simulations. Later, in [93], this is tested in mainly a suburban environment yielding a standard deviation error of 3.4 m for a 12-min run. Yang et al. estimate the baseline between the vehicles using a weighted least squares algorithm and weighting each pseudorange according to the received carrier-to-noise ratio in [94]. Experimental results with the static 3 m and 8 m baseline on a rooftop yield baseline length errors of up to 40 m. De Ponte Müller et al. demonstrate that by exchanging GNSS pseudoranges between the vehicles, an unbiased relative position estimate is obtained at the expense of increasing the noise [95]. With this method, the distance between the vehicles can be estimated with less than 0.80 m and 1.30 m accuracy in highway and urban environments, respectively. A technique in which the pseudorange biases are estimated in a cooperative way between the vehicles is presented by Lassoued et al. in [96]. A horizontal relative positioning error of 2.46 m is achieved. However, pseudorange bias estimation is a slow process that takes above ten minutes to converge.

#### 4.2.4. Differential Carrier Phase Ambiguity Resolution

A number of groups have addressed the relative positioning problem of vehicles by solving differenced GNSS carrier phase ambiguities rather than using GNSS differenced pseudorange techniques. The potential of this approach comes from the fact that noise in the Phase-Locked Loop (PLL) of the GNSS receiver is smaller by several orders of magnitude [58]. In order to determine the range towards the satellite, the integer number of cycles has to be resolved. This task is especially difficult in vehicular environments due to signal disturbances, satellite blockage and multipath, which lead to cycle slips that will reset the resolution algorithm.

Besnayake et al. built a test platform for relative positioning yielding range errors of less than 1 m 99% of the time in open sky and 90% on obscured roads in [97]. Hwang et al. propose a particle filter design that samples from the relative position domain to solve the carrier phase ambiguity [98]. The use case they are focusing on is relative positioning of low orbit satellites. Travis et al. have worked on a trajectory duplication using carrier phase-based relative positioning in [99,100]. Ansari et al. have investigated cooperative relative positioning exchanging Real-Time Kinematic (RTK) position solutions between vehicles [101]. Further on, they looked at integrity concepts based on the same methods in [102]. By exploiting the redundancy when integrating measurements from different vehicles, Luo et al. have shown that the time to integer fixing can be decreased at the same time that the reliability is increased [103]. De Ponte Müller et al. conclude that it is possible to fix ambiguities with single-frequency, low-cost receivers in benign road environments and to obtain a centimeter-level relative positioning in [104]. While the previous work output the relative position in Earth-fixed coordinates, Zeng et al. propose a GNSS carrier phase-based ground truth system for evaluating the performance of radar sensors [105]. They conclude that centimeter-level accuracy quickly degrades when less than four satellites are in view of both vehicles.

GNSS carrier phase relative positioning offers extremely accurate full three-dimensional relative position and relative velocity at the output rate of a GNSS receiver. If coupled with inertial on-board sensors, the update rate can be enhanced to meet the requirements of safety-critical applications. The main disadvantage is its sensitivity and, consequently, the limited continuity in its performance. Trees, bridges and highway gantries cause momentary drops in the signal level, loss of lock on the carrier phase and cycle slips. Low cost receivers are especially sensitive, and a continuous tracking of the carrier phase is practically impossible in usual scenarios.

### 4.3. Coordinate Frame Transformation

**Figure 4:**
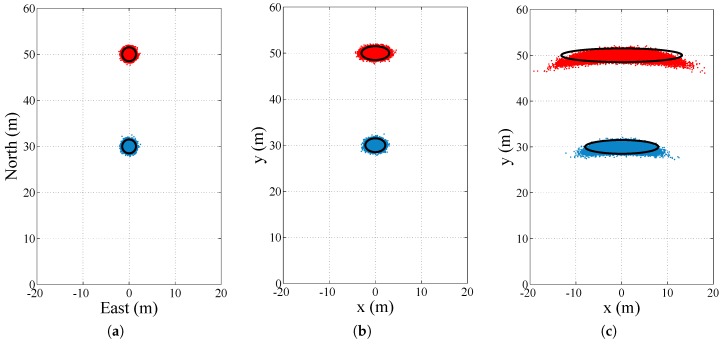
The figures show the uncertainty in the relative position of a vehicle driving 30 m (blue) and 50 m (red) ahead. The red and blue dots are the particle clouds, and the black ellipses are the linearized Gaussian uncertainty. Figure (**a**) shows the relative position in the navigation frame coordinates with a 0.5 m (1σ) error in the north and east direction. After rotating this position into the ego vehicle frame using the ego vehicle’s heading angle with an uncertainty of 1${}^{\circ }$ (1σ) in Figure (**b**) and 5${}^{\circ }$ (1σ) in Figure (**c**), the uncertainty in the relative position is clearly increased.

Cooperative approaches are mainly based on GNSS positioning information, which is always referenced to an Earth-fixed coordinate frame. Ranging sensors, on the contrary, output the relative position information of another vehicle in an ego vehicle reference frame. Since the ego vehicle motion control is also executed in this frame, it makes sense to translate the relative position of other vehicles from the Earth-fixed frame to the ego vehicle reference frame when using a cooperative approach. When considering a two-dimensional relative position and two vehicles driving on the same plane, the ego vehicle’s heading is required to rotate the absolute position of the target vehicle into the ego vehicle’s reference frame. This is displayed in Figure 4 for two target vehicles located at a 30 m (blue) and a 50 m (red) distance in front of the ego vehicle, respectively. Figure 4a shows the relative positions in an east-north coordinate frame with an estimation error of 0.5 m (1σ). Figure 4b,c show the relative position in the ego vehicle reference frame after a rotation with the ego vehicle heading angle, which has an error of 1° and 5°, respectively. A higher relative position uncertainty is the result after performing the coordinate frame translation.

Thus, it is crucial to have an accurate estimate of the heading of the ego vehicle for accurate 2D relative position. GNSS-based velocity information is able to offer heading information below 0.25 ${}^{\circ }$ at a higher driving speed and good satellite signal conditions [106]. Henkel et al. obtain heading accuracies of 0.5${}^{\circ }$ (1σ) by using a GNSS carrier phase resolution algorithm with a one-meter separated antenna setup in [107]. For bridging GNSS outages and sections of slow driving, sensor fusion techniques with inertial sensor and/or steering angle sensor information are required [108].

### 4.4. V2V Communication Performance

The presented cooperative approaches are based on the exchange of position, velocity and orientation information between the vehicles. This exchange is performed by using V2V communication. The current standard for direct wireless inter-vehicle communication in the U.S. and Europe is based on the IEEE 802.11 standard working in the 5.9 GHz band. Further details on this communication technology can be found in the corresponding standards, [109,110], and in the survey papers [111,112,113].

Under ideal line-of-sight conditions, communication between vehicles above 1 km is possible with this technology [114,115]. However, in real-world environments, adverse radio-frequency propagation conditions, including shadowing, blockage, multipath propagation and high vehicle dynamics, causing large- and small-scale fading, will make it difficult to achieve the message update rate required by the safety application. Many research groups have investigated the radio propagation channel in vehicular environments [116,117,118,119]. Other research groups look into the impact in terms of the attenuation of certain obstacles, such as large vehicles on the V2V signal [120,121]. The overall communication performance in terms of packet error rate and effective communication range is the object of research in [114,122,123]. According to Boban et al., on average, an effective V2V communication range up to 400 m can be achieved in highway environments, while only a 100 m reliable communication range can be expected in urban scenarios [123].

Additionally, the radio channel is shared among different traffic participants. Carrier Sense Multiple Access with Collision Avoidance (CSMA-CA) is a random contention protocol that makes it possible for different nodes to communicate over a shared medium while minimizing the number of packet collisions. However, in high-traffic scenarios with high communication load, the number of packet collisions increases dramatically [124,125].

The challenges for both the physical propagation conditions and the medium-access technology are the cause for an unreliable information exchange between the vehicles, which leads to a decreased awareness of the ego vehicle regarding the relative position of surrounding vehicles. A suitable metric for this awareness is the update delay [126] or packet inter-reception time [127], which measures the probability of experiencing a communication outage from a certain transmitter longer than a given time. During such communication outages the relative position of a target vehicle has to be predicted, increasing the uncertainty and the risk in the case of a sudden event, such as harsh braking or a strong change in direction.

## 5. Sensor Fusion for Relative Vehicle Positioning

As we have seen, every relative positioning technology for vehicles has its advantages and disadvantages regarding its performance in terms of accuracy, reliability, availability, range or field of view. Consequently, an intelligent strategy is to combine the information from different sensors and localization technologies in order to obtain a more robust estimation of the target vehicle’s position. This is usually accomplished by applying sensor fusion techniques, where the information is combined in a way that the resulting estimation uncertainty is lower than the uncertainty of each of the information sources individually. Ideally, the limitations of one type of sensor are overcome with the strengths of a complementary sensor. For instance, a common approach is the fusion of radar sensors and cameras. The limited sensing range of camera systems and the absence of range rate measurements can be enhanced with the use of a radar sensor, which, on the other hand, profits from the good lateral resolution and feature richness of camera systems. This approach has been followed by Kato, Broggi and Bombini in [128,129,130] using monocular cameras. The combination is found to increase reliability in vehicle detection and overall robustness of the relative positioning system. Wang et al. achieve a 92% detection rate with no false alarms with a two-stage fusion of mm-wave radar and monocular camera in [131]. A stereo-camera system is combined with two radar sensors by Cesic in [132]. As explained in [133,134], a radar sensor can profit from the precise lateral accuracy of a laser scanner, while contributing with an accurate relative velocity estimate of the target vehicle and a higher robustness against climatological adversities. A multi-sensor platform consisting of vision and LIDAR has been presented in [135]. The authors acknowledge that although an increased detection rate and correct target classification are accomplished, bad weather conditions affect both sensors equally. All non-cooperative approaches, i.e., radar, laser scanner and camera systems, are fused by Walchshäusel et al. for collision mitigation by autonomous braking in [136]. Cho et al. evaluated a two-layer sensor-fusion platform consisting of 14 asynchronous on-board sensors, including radar, LIDAR and vision cameras [137]. They claim a 93.7% detection rate with 5.7 false positives per minute. Vu et al. present a sensor fusion approach for detecting obstacles and other vehicles based on radar, laser scanners and cameras [138]. Among the challenges faced when combining different perception sensors, there is the need to synchronize adequately the various measurements and to correct for the displacement due to the mounting location on the vehicle.

An intelligent approach is to take advantage of the complementary nature of on-board ranging sensors and cooperative approaches based on GNSS and V2V communication. Cooperative positioning can help on-board sensors in different ways. On the one hand, it makes it possible to identify target vehicles and to track them over time, even when disappearing behind other vehicles or obstacles. It is inherent to a cooperative approach based on CAM or BSM over V2V that each vehicle has a unique identifier. Further on, a cooperative approach can extend the perception range of the on-board sensors behind curves or crests in rural areas. Even in urban environments, CAM from vehicles behind buildings at intersections can be successfully received. Additionally, a cooperative approach will make it needless to equip a vehicle with multiple sensors to get an all-around view of the surrounding situation. On the other hand, since on-board ranging sensors have, in general, better accuracy than GNSS-based cooperative solutions, they can stabilize the relative position estimation during GNSS outages, as for instance in tunnels or against multipath propagation in urban canyons. Furthermore, communication outages due to shadowing, line-of-sight obstruction or packet collisions can be overcome with on-board ranging sensors. In this way, a cooperative/non-cooperative solution is able to offer higher availability and continuity with an increase in accuracy for safety critical applications.

**Figure 5:**
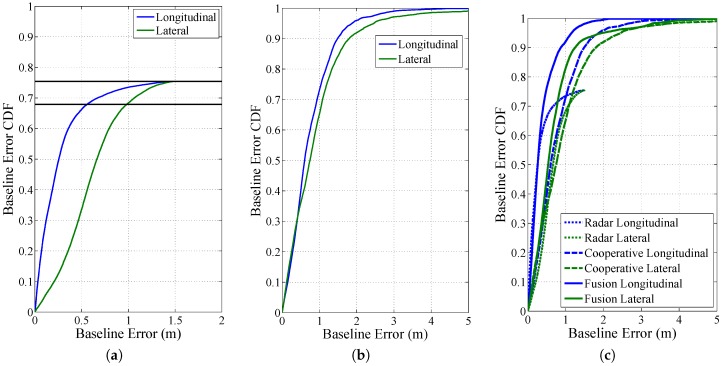
The figures show the relative positioning error in longitudinal (blue) and lateral (green) direction for a radar sensor (**a**), a cooperative approach (**b**) and the fusion of both (**c**) while driving on a highway [114]. The radar sensor has a good longitudinal accuracy, but a limited availability of 75%. The cooperative approach has a larger error, similar in both directions and continuous availability. The continuous lines in (**c**) show the fusion of both, where an increased precision and availability are achieved.

Unfortunately, regarding the fusion of on-board ranging sensors and cooperative approaches based on V2V, only a few publications exits. Röckl et al. have worked on augmenting the capabilities of radar sensors with absolute positions exchanged over V2V communication in [26,139]. They proved an increase in availability with their approach in experimental measurements. A simulative evaluation of a multi-target tracking approach based on an automotive radar sensor is presented in [89]. Obst et al. suggest a plausibility checking of CAM via a monocular camera in [13]. De Ponte Müller et al. proposed a sensor fusion framework, where cooperative relative positioning is supported by a radar sensor in [114]. This setup was tested in both highway and rural environments showing that the cooperative approach profits from the higher accuracy of the radar sensor, whereas the radar sensor’s poor availability due to obstructions is enhanced by the availability of V2V communication. Figure 5 shows the accuracy in the relative position in both the longitudinal (blue) and lateral (green) direction on the highway. Figure 5c demonstrates that an increased availability and accuracy is achieved when compared to each solution alone. In [140], Fujii et al. performed a simulative evaluation of an intersection where vehicles were possibly equipped with ranging sensors and/or V2V communication. They showed that by equipping vehicles with V2V communication, the average cooperative positioning accuracy could be increased. Wang et al. propose in [141] a decentralized filter that fuses inter-vehicle UWB ranges with GPS-based observations from each vehicle that achieves sub-meter relative positioning accuracy.

## 6. Conclusions

This survey paper has presented a review on different cooperative and non-cooperative relative positioning sensors and technologies for driver assistance systems in current vehicles and future automated vehicles. Table 4 summarizes all presented relative positioning techniques. The requirements reviewed in Section 2 are added in each column to evaluate qualitatively each of the techniques according to the analysis of the previous sections. Five symbols (+ +, +, ∘, −, − −) are used to symbolize if a ranging system performs positively, neutrally or negatively in a certain category.

On-board ranging sensors, as radar sensors and laser scanners, offer a high accuracy in their range estimation. While radar sensors have poor lateral resolution, laser scanners can calculate the lateral distance with higher accuracy. Both have an acceptable update rate for safety critical applications (above 10 Hz), but only radar sensors offer a direct estimation of the relative velocity. Vision-based systems, based on stereo cameras, are only able to estimate the distance to vehicles in the near range and have to use additional information about objects and context to estimate the distance to more distant vehicles. Relative speed can only be estimated by looking at consequent images. Laser scanners and camera systems have lower availability since they rely on visible light and are thus sensitive to adverse lighting and climatological conditions. Radar sensors, eventually supported by laser scanner or cameras, are a very suited approach for relative positioning for safety-critical advanced driver assistance systems. Radar and vision-based solutions correctly complement each other regarding longitudinal and lateral performance. By moving towards higher frequency bands, increased bandwidth for radar sensors will become available, which leads to a higher degree of detail in the echoed signal. Future research will provide more robust and precise detection and tracking algorithms for radar sensors. Furthermore, image processing algorithms for vision-based vehicle tracking systems will continue evolving to decrease false detection rates and misclassification of road objects.

Regarding cost, radar sensors have dropped in price in the last decade. The same is expected to happen to vision-based systems, since camera technology has found its place in the consumer market and the technology has matured for introduction into the automotive segment. Laser scanners, with its mechanical parts, will probably need some more time to be attractive enough to find market introduction.

All non-cooperative approaches have line-of sight characteristics and are easily obstructed by obstacles, such as other vehicles, or have a limited range in curvy rural roads or urban environments. Furthermore, cooperative transponder-based approaches using RSS, RTD and TOA measurements exhibit too large errors when in non-line-of-sight conditions. This is considered an important drawback for vehicular safety applications that need to react in a timely manner to dynamic events ahead of the vehicle immediately in front. For this reason, cooperative approaches based on V2V communication, which can cope with occlusions of the line-of-sight up to several hundreds of meters, offer a real advantage. Relative positioning is achieved by the exchange of positioning information between vehicles. Here, different solutions compete with each other to meet the requirements of advanced driver assistance applications. Standalone GNSS solutions do not meet the requirements on accuracy and availability on relative position and relative speed. The fusion of GNSS with on-board kinematic and inertial sensors for absolute position determination increases both the availability and the accuracy. Centimeter-precise relative position can be achieved with GNSS carrier-phase solutions, but has the drawback of high sensitivity to satellite line-of-sight obstruction causing a limited availability and being only reserved to open-sky scenarios. Nevertheless, the limited availability and the low accuracy of GNSS-based solutions in challenging environments, such as urban canyons or tunnels, are still the main issues that need to be addressed in the future for cooperative approaches based on V2V communication. Highly precise maps will be the key to localize accurately future autonomous vehicles in absolute coordinates. By using their on-board perception sensors, autonomous vehicles will be able to recognize surrounding features and either localize themselves or share these features with other vehicles in order to position themselves in relation to others.

The fusion of both cooperative and non-cooperative approaches yields the most promising relative position estimation performance. It is suggested to combine the high accuracy and good robustness against the lighting and climatological conditions of radar sensors, with the extended all-around range and identification capabilities of V2V communication. Vision-based systems and radar sensors could in the future incorporate the information on surrounding road users provided by a cooperative technology at the lowest-level to improve vehicle detection and the resolution of different targets. For a cooperative approach, it is envisioned that the exchange of GNSS-derived pseudorange and carrier-phase measurements for differential positioning along with kinematic and inertial sensor information will provide the highest accuracy, availability and robustness.

## Figures and Tables

**Table 1:** Radar sensors.

Sensor	Frequency	Bandwidth	Range	Azimuth Angle	Accuracy	Cycle
Bosch LRR3	77 GHz	1 GHz	250 m	±15${}^{\circ }$	0.1 m, 0.12 $m\;s^{-1}$, -	80 ms
Delphi ESR	77 GHz	-	174 m	±10${}^{\circ }$	1.8 m, 0.12 $m\;s^{-1}$, -	50 ms
Continental ARS30x	77 GHz	1 GHz	250 m	±8.5${}^{\circ }$	1.5%, 0.14 $m\;s^{-1}$, 0.1${}^{\circ }$	66 ms
SMS UMRR Type 40	24 GHz	250 MHz	250 m	±18${}^{\circ }$	2.5%, 0.28 $m\;s^{-1}$, -	79 ms
TRW AC100	24 GHz	100 MHz	150 m	±8${}^{\circ }$	-, -, 0.5${}^{\circ }$	-

**Table 2:** Laser scanners.

Sensor	Dimensional Resolution	Range	Azimuth Angle	Accuracy	Cycle
Quanergy M8-1	3D	150 m	360${}^{\circ }$	0.05 m, -, 0.03${}^{\circ }$	33 ms
Ibeo LUX	2D	200 m	110${}^{\circ }$	0.1 m, -, 0.125${}^{\circ }$	20 ms
Continental SRL1	2D	10 m	27${}^{\circ }$	0.1 m, 0.5 m/s, -	10 ms
Velodyne HDL-64E S2	3D	120 m	360${}^{\circ }$	0.02 m, -, 0.09${}^{\circ }$	50 ms

**Table 3:** Time-of-flight cameras.

Sensor	Resolution	Range	Azimuth Angle	Accuracy	Cycle
PMD CamBoard	200 × 200	7 m	40${}^{\circ }$	-, -, -	60 fps
PMD CamCube	200 × 200	-	-	-, -, -	-
SwissRanger SR4000	176 × 144	10 m	40${}^{\circ }$	1 cm, -, -	50 fps

**Table 4:** Relative positioning techniques.

Relative Positioning Technique	RSS Ranging	Radar	Laser Scanner	ToF Camera	Vision Based	RTD	GNSS Only	Differential GNSS	Diff. GNSS + INS	GNSS Carrier Phase
Cooperative	No	No	No	No	No	Yes	Yes	Yes	Yes	Yes
Accuracy	-- 5–20 m	++ 0.1 m 0.2 $ms^{-1}$ 0.1${}^{\circ }$	++ 0.02 m 0.5 $ms^{-1}$ 0.1${}^{\circ }$	++ 0.1 m 0.2 $ms^{-1}$ 0.1${}^{\circ }$	− 1–5 m	+ 0.5–1 m	− 2–10 m 0.01 $ms^{-1}$ 0.25–10${}^{\circ }$	∘ 0.5–5 m 0.01 $ms^{-1}$ 0.25–10${}^{\circ }$	+ 0.5–2 m 0.01 $ms^{-1}$ 0.25–1${}^{\circ }$	++ 0.01–0.05 m 0.01 $ms^{-1}$ 0.25–10${}^{\circ }$
Availability	∘	∘	∘	−	∘	+	−	−	+	--
Reliability	∘	++	+	∘	∘	∘	+	++	++	+
Range and Field of View	++ 400 m 360${}^{\circ }$	∘ 250 m ±15${}^{\circ }$	∘ 200 m 360${}^{\circ }$	-- 20 m 40${}^{\circ }$	− 40 m ±20${}^{\circ }$	++ 300 m 360${}^{\circ }$	++ 400 m 360${}^{\circ }$	++ 400 m 360${}^{\circ }$	++ 400 m 360${}^{\circ }$	++ 400 m 360${}^{\circ }$
Dimensional Resolution	-- 1D	∘ 2D	+ 2D/3D	+ 2D/3D	+ 2D/3D	-- 1D	+ 3D	+ 3D	+ 3D	+ 3D
Target Resolution	--	∘	∘	+	+	++	++	++	++	++
Non-Technical	++	∘	−	--	+	++	++	+	−	−

## Abbreviations

The following abbreviations are used in this manuscript:

ACC	Automatic Cruise Control
CAM	Cooperative Awareness Message
ETSI	European Telecommunication Standards Institute
DGPS	Differential GPS
FCA	Forward Collision Avoidance
FMCW	Frequency-Modulated Constant Wave
GNSS	Global Navigation Satellite System
GPS	Global Positioning System
INS	Inertial Navigation System
ITS	Intelligent Transportation Systems
ISM	Industrial, Scientific and Medical
LCA	Lane Change Assistant
LIDAR	Light Detection and Ranging
TOF	Time-Of-Flight
RSS	Received Signal Strength
RTD	Round-Trip Delay
SAE	Society of Automotive Engineers
UWB	Ultra-Wideband
V2V	Vehicle-to-Vehicle
